# Caring for/with Modernist Playthings: Fidgeting with Objects in Tennessee Williams’s *The Glass Menagerie*

**DOI:** 10.1007/s10912-024-09848-y

**Published:** 2024-05-29

**Authors:** Ishita Krishna

**Affiliations:** University of York, York, UK

**Keywords:** Literary and Theatrical Objects, Modernism, Realist Drama, Theater and the Nonhuman, American Realism, Feminist Criticism, Disability Studies

## Abstract

Modernist literature of the early to mid-twentieth century on both sides of the Atlantic is replete with examples of a particular kind of relationship with objects, namely, the touching, collecting, and grasping of small, often highly personal, and ostensibly quotidian objects. From John’s glass collection in Woolf’s “Solid Objects,” Peter Walsh’s stroking of his pocket-knife in *Mrs. Dalloway,* Miriam’s frenzied absorption with flowers in Lawrence’s *Sons and Lovers*, to Laura’s fiddling of her glass menagerie in Tennessee Williams’s eponymous play, fidgeting in modernist literature and drama reveals a particular tendency of not just characters’ possession *of* things but also their possession *by* things. This phenomenon, I argue, allows characters to practice care as they withdraw from oppressive narratives of normalcy and (economic and biological) productivity, challenging their exclusionary and othering configurations. My paper looks at fidgeting in *The Glass Menagerie* as a part of this larger ideological and haptic orientation in modernist literature. The care invested by Laura in her intimate relationship with these “playthings” allows her to intercept not only male narrativizing forces and articulation of herself but also the rhetoric of productivity that circulates both within the play and in the larger economic backdrop of post-depression America. My paper attempts to foreground these objects of care in our readings of the play and modernist texts in general and, in so doing, highlight their importance as lenses of analysis that render visible alternate forms of agency and resistance. Lastly, it attempts to reframe fidgeting as an act of embodied refusal, evoking the radical potential of refusal within feminist and disability studies.

## Introduction

Tennessee Williams pointedly situates his play *The Glass Menagerie* in the Great Depression, after the economic crash of 1929. This post-Gatsbian landscape harbored suspicion towards the myth of the American dream and the promise of never-ending economic prosperity. This sense of realization of the unsustainability of the consumerist, mass production model was also accompanied by a transformation in American drama’s relationship with the real. We see a deliberate change not only in the abstract conception of dramatic representation’s approach to the real but also a material and physical evidence of this transformation, the latter becoming both the symptom of this change and also writing a material manifesto of a new kind of theater. This can be seen in new directions within theater making, scenography, and playwriting with the advent of American Experimental Drama (Beard 2008), new American Stagecraft (Doona 2012), and “Plastic theatre,” Williams’s version of poetic realism (Williams 2016, 5). Theatrical objects on this new American stage of the early mid-twentieth century impose their presence as products of creation, fiction, and consumption simultaneously. This tendency, rooted in the din of mass production and consumerism of early twentieth-century America, paints characters’ encounters with objects in a color that is symptomatic of a rapidly changing relationship with material reality both within and outside the theater.

It is in this economic, political, social, and theatrical backdrop that Tennessee Williams’s *The Glass Menagerie* situates itself, and it is this nexus of forces that refract and reflect from the fragile, ephemeral but firmly present objects and materials that litter the textual and stage space of the play. The play recounts the story of the Wingfield family with the mother’s desperate search for a husband for her daughter, the brief encounter with a gentleman caller, and the daughter’s fidgeting with her glass menagerie and victrola, narrated by the son before he, like his father, abandons the family. Offered as a memory, the different elements of the play, both human and nonhuman, fight back or cave in not only against the outside forces of the period but also against the forces of narrativizing itself and of absorption into Tom’s memory. If a text’s relationship with the real dictates genre, in this play, the relationship has as its fulcrum the “delicate or tenuous material” (Williams 2016, 5) of memory that constantly questions itself, thus forging a second-guessing, self-doubting brand of realism that stops short of self-reflexive. The play’s tenuous relationship with the real and the generic preoccupations, I argue, have much to do with the network of objects that layers, often uncomfortably, the Wingfields’ family network. The generic battle is then played out in the quintessentially realist domain, the living room of Wingfields’ house, with the prominent objects, mainly the glass but also flowers and the victrola, as the weapons.

The narrative, psychological, and sensory implications of the alternative fidgeting with these objects are by no means only generic but also structural, visual, affective, and even hermeneutic: taunting established readings of the play, particularly those of its ambiguous ending. I conceive the specific object encounter, fidgeting, as an umbrella term covering a wide range of impulses and orientations towards objects like fondling, caressing, constant fiddling, grasping, collecting, and at times, frenzied, obsessive, or desperate clinging on to objects. I offer fidgeting as an important lens for modernist object encounters by unveiling its complex operation in Williams’s play and, in so doing, proposing the need to engage in its study to rescue the specific encounter and phenomenon from relative discursive neglect in literary and theater research. The specific iteration of this approach to objects in *The Glass Menagerie*, I will argue, underpins a practice of care by virtue of the very nature of the gesture, highlighted further by its positioning among “uncaring” encounters and materials.

Through the exploration of fidgeting in the play, I hope to propose the overlooked centrality of this object encounter as both a generative subject and lens of analyses, drawing from the larger haptic and sensory orientation towards fidgeting in modernism. This reconsideration of the play is not to erase its problematic elements, particularly with regard to its representation of disability, but to use this overlooked lens in modernism to highlight the representation of the self, the (nonhuman) other, and processes of othering in the play. While I will trace fidgeting through the play more generally, I will pay particular attention to Laura’s glass objects and their fidgeting as a performance of care, exploring in the process how material care allows Laura to withdraw from uncaring narratives of compulsory (re)productivity, normalcy, and male storytelling. Objects of care help Laura perform alternate forms of agency and forestall the othering forces of established value systems. Looking at fidgeting as material care problematizes critical approaches that dismiss Laura as a passive object of care, allowing to reposition her as an agential subject or carer instead. Lastly, I reframe Laura’s fidgeting as a practice of care and a mode of refusal, evoking wider resonances of gendered work and passivity within feminist and disability studies.

## Laura(’s) Objects

The dramatic action of *The Glass Menagerie* is sparse, as Harold Bloom argues, with only two main lines of thought: Tom’s desire to escape and Amanda’s fixation on finding Laura a husband (Bloom 2007, 20). The arrival of the gentleman caller is the central dramatic event against which the structure of the play is organized: Part 1, “Preparation of the Gentleman Caller,” and Part 2, “The Gentleman Calls.” In both these conceptions of *The Glass Menagerie*’s structure, Laura emerges as a passive recipient of others’ care, desires, and decisions or, in other words, the *object* of care and action rather than an agent thereof. Laura is described through her relationship with fragile objects: she toys with her glass collection, skips her business college and typing course to look at tropical flowers, and winds the victrola in moments of intensity and doubt. Her absorption with glass evokes frustration in others who are eager to find her a place within the familiar scripts of the conventional marital plot and (reproductive or marketable) productivity. Amanda’s frustration is clear in scene 2: she chastises her daughter backed by the screen image of a “swarm of typewriters” and asks, “So what are we going to do the rest of our lives? … Amuse ourselves with the glass menagerie, darling? Eternally play those worn-out phonograph records your father left as a painful reminder of him?” (Williams 2016, 20). This echoes critical approaches to Laura that equate her passivity to victimhood and inability. Laura’s resistance to the world of typewriters with its promise of a business career and attraction to objects that mark absences instead, the transparent glass and the records left by an absent father, comes with the looming threat of becoming one of the “little birdlike women without any nest—eating the crust of humility all their life!” (20).

Laura’s relationship with the glass menagerie has been read variously as liberating or restrictive, a sign of her difference, anxiety, or debilitating shyness (Babcock 1999, 24), or a metaphor for the trap of modern existence (Nagpal 2016, 35). Critics list a number of negative sources and implications of Laura’s incessant fidgeting with the glass animals or the victrola, arguing that it forecloses the possibility of action and becomes an instrument of difference or escapism (Bigsby 1997; Kent 1988). Pragya Gupta (2016, 161) contends that the text asks, “Is the glass menagerie a canvas for Laura to fashion herself as an artist? Or is it merely an alibi for beautifying and embroidering a victim complex?” While these critics often agree that the play’s commentary is directed against the systems that victimize Laura and the emissaries of those worldviews, there is nonetheless a tendency to see the play as a story of a “woman’s wasted life,” casting Laura in the mold of “the poor crippled girl—forever a child playing with her glass animals” (Fox 2016, 137), overlooking that we are reading Tom’s narration of her. This Laura-as-victim critical camp reproduces the image of Tom’s articulation of Laura and fails to read through the gaps and splinters in Tom’s memories that are largely inaccessible through a subject-oriented lens. By exercising inactivity within the dramatic form, which is based on action and temporality, Laura’s object encounters create an alternate space away from Tom’s narrative, allowing us to access the gaps and slippages in Tom's memory and problematize the critical and narrative victimization of Laura.

Laura’s glass animals have also been seen as the reasons for her “inadequacy, self-indictment, and aversion… [due to her] commercial and biological unproductivity” (Gupta 2016, 164). This view equates agency to action, resistance, and voice, eliding over Laura’s own brand of agency that she performs *through* objects and not *despite* them. The stage directions describing her interaction with the glass animals—“She reaches quickly for a piece of glass” (Williams 2016, 21); “Laura takes the glass uncertainly turns in her hands a piece of glass to cover her tumult” (74)—portray an uneasy, frenzied gesture that precedes the grabbing and fidgeting with the little objects, reflecting more the restlessness of a parent separated from their infant and less inadequacy and victimhood. Williams himself makes Laura’s identification with the glass animals clear, as she is “like a piece of translucent glass touched by light, given a momentary radiance, not actual, not lasting” (50). It might be easy to argue then that the glass is her surrogate self as a fragile, yet beautiful, cared-for object exhibited for others. But while the very things that preserve her creative impulses might seem to insulate her from meaningful connections with others and the present, they do not make her inert. As Pragya Gupta (2016, 165) argues, “While she is crippled and dependent in the outside world, she is the guardian and nurturer in at least this one relationship, she is the artist figure here.” Laura, too, defines her fidgeting as a skilled embodied practice of care, recognizing that “Glass is something you have to take good care of” (Williams 2016, 74). She seems to invertedly reproduce her relationships with Tom and Amanda, where she is forever frozen in an infantile state of a delicate object of care. With her glass objects, she appropriates the role of the carer and the agency of one. The tactile act of fidgeting then emerges for Laura as a vehicle for occupying an agential subject position, or in other words, the objectifying nature of the material and tangible care allows her to occupy the space of a subject heretofore uninhabitable.

Another common critical approach is reading fidgeting as an escapist tic or a distraction, a retreat to a fictive, mythic world away from the present in the vein of Tom’s nightly escapades or Amanda’s nostalgic tales of her Southern belle past (Nagpal 2016). But unlike Tom’s poetic and adventurous pretensions that mistake “movement for progress” (Bigsby 1997, 38) and Amanda’s delusional hopes of reliving her past glory, Laura’s fidgeting, I argue, is marked by a knowing resignation that both the myth of the past and “cruel optimism” (Berlant 2011) for the future have failed her, given their intolerance for “the artist, the dispossessed, or the abandoned” (Bigsby 1997, 32). The act of material care creates and anchors her firmly in an alternate present marked by an acknowledgment of the failure of both personal and national myths.

## Laura Prefers Not to: Material Care and (Un)action

A person like Laura would then find herself alienated not just from the bourgeois patriarchal morality but also from the fundamental forces of what Bigsby (1997, 33) calls the American “national myth,” born from the rhetoric of unrelenting consumerism and progress. Her fidgeting acquires specifically American valency in the play, lodged as it is within the context of the American dream of perpetual progress and material success. Beyond being an expression of the inner landscape of a character, a thematic, generic, or narrative metonym, or a gesture inherited from past theatrical/literary traditions, the personal gesture is also invaded by the economic and political inflections and the kind of commodity fetishism that characterized the mass-producing, industrial economy. Laura ruptures this fetishistic approach to objects by developing a deeply personal relationship with her glass animals that replaces objects’ economic value with personal value. In caring for objects without exchange value, utility, or function (in stark contrast to the mass-produced shoes at the factory where Tom works and his functional and impersonal approach to them), she forestalls and even replaces the objects’ functional value, investing them with personal value. Furthermore, this practice of care evokes larger questions of gendered care, which within systems of compulsory economic productivity is invisibilized and unrecognized as valuable or productive work (Chen, Khúc, and Kim 2023, 2). Laura’s objects of care then rehearse the “everyday form of inaction, inscrutability, and non-productivity” (Mengesha and Padmanabhan 2019, 2) that emerge from a refusal of productivity. The material stubbornness of this object encounter defies clear logic, motive, or rationale within existing structures and resists alignment with an easily discernible symbol, projected abstraction, or external signifier. By refusing intelligibility or hermeneutic closure within outside structures (economic and semantic), the gesture reveals the inadequacy of these systems, as I will explore later.

The Wingfields’ desires and demands for Laura’s life reflect at least partly the narratives circulating in 1930s America. These comforting myths of unending progress not only are based on the homogenization and standardization that render everything and everyone interchangeable (people are reduced to consumers and objects to commodities) but also alienate and persecute what Babcock (1999, 18) terms the “freaks,” that is, anyone who resists their role in the rhetoric of productivity. Bigsby (1997, 35) has pointed out that, within the play, “there is a powerful sense not merely that the animating myths of America have failed those who look for some structure to their lives, but that those myths are themselves the root of a destructive materialism or deceptive illusion.” Laura’s character reflects how she has been failed by these myths of normalcy that perpetuate compulsory productivity and heteronormativity. Fidgeting as a material resistance to these myths then becomes an embodied expression of this failure.

Ann M. Fox argues, “The play looks not only to the system which sees her as extraneous, but particularly embodies her experience as a disabled woman in a society obsessed with compulsory ablebodiedness. It is an experience that is inextricably intertwined with the compulsory heteronormativity that seems to more obviously oppress her in the shape of her mother’s constant matrimonial ‘plans and provisions’ for her” (Fox 2016, 137). The national myths of productivity and normalcy then further alienate Laura whose “childhood illness has left her crippled, one leg slightly shorter than the other, and held in a brace” (Williams 2016, 3). Amanda voices the prescriptions of normalcy that infiltrate the Wingfield living room in her frustration and desperation to “standardize” her children: “Why can’t you and your brother be normal people?” (55). The compulsory heteronormativity here is inseparable from compulsory ablebodiedness within the cultural urgencies to “be normal.” Fox (2016, 135) further argues that “Williams is damning those myths of normalcy circulating at the time, ones that particularly singled out both disabled and queer bodies for persecution.” These forces then not only alienate resisting bodies but systematically overlook, eliminate, and redefine outliers: disabled, unproductive, queer bodies.

Laura is constantly placed within the stock disability stereotype of the “asexual, dependent, a perennial child” (Gay 2008, 3) by her family. This can be seen in Laura’s reductive casting as an object of care, as her disability and passivity are read as an invitation to subject her to their paternalistic and victimizing rhetoric. She even notices Amanda’s eagerness to perform the role of a selfless caregiver: “Mother, when you’re disappointed, you get that awful suffering look on your face, like the picture of Jesus’ mother in the museum!” (Williams 2016, 20) Laura is then cared for on others’ terms and an object of their paternalistic care. As an (unwilling) object of care, Laura finds herself further alienated from the value system of her carers, who equate passivity with victimhood and agency with action.

Laura’s fidgeting then registers a refusal to work by someone who has been refused *as* a worker or an agent of action. Mel Y. Chen, Mimi Khúc, and Jina B. Kim (2023, 3) contend that “disability’s relationship to labor has … been one of exclusion.” Disability becomes conflated with the absence of productivity, which is used as a justification for devaluing and objectifying bodies. (This projected absence overlaps with the invisibilization of gendered practices of care discussed above.) Laura’s fidgeting as embodied refusal of productivity is arguably itself read as disability (as explored earlier, she is incessantly victimized), given that with the rise of the industrial period, disability began to describe the inability to work and produce at socially necessary rates (Chen, Khúc, and Kim 2023, 3; Gleeson 1999, 100).

Objects become a mode of performing care and, by extension, agency for Laura as she alternatively occupies the position of an intentional subject and a passive plaything, consciously or otherwise. Equally, Laura’s alternate form of agency and subjecthood are *object-like.* In her (perceived) passivity, she practices what Eunjung Kim calls “object-becoming,” wherein “embodying objecthood, surrendering agency, and practicing powerlessness” offers a challenge to the “exclusionary configurations of humanity that create otherness” (Kim 2015, 295). Laura plays with the subject-object dichotomy to exercise a brand of agency that leaves her vulnerable to patronizing advice, paternalistic dismissal, critique, and even derision from the ambassadors of the world of action: Amanda and, later, Jim. At the same time, this allows her to turn away from the industrial-patriarchal nexus and the bourgeois social scripts of marriage, love, and productivity as also “ability-based determination” (Kim 2015, 296) of her legitimacy and value.

While she ostensibly seems to be inert, ineffectual, and a passive recipient of other’s care and demands for her life in her Hamlet-like resistance to action, she appropriates the role of the carer, the fidgeter, and the agential subject by reframing the very idea of agency where it is no longer reliant on action. In practicing material care through fidgeting and her own *objectness*, she turns to a world not of inactivity or lack of action but a deliberate absenting of action, or what I call rebellious (un)action or unproductivity- the interim moments and liminal spaces of stasis, silence, and respite from the rhetoric of productivity and the unfolding of time, narrative, and the conventional closure of realism. Performing the unaction of care allows Laura to resist the outside forces, both fictional forces of absorption into the male narrative and socio-economic forces of marriage and the production imperative. To paraphrase the title of Mengesha and Padmanabhan’s (2019) work, through the unaction of fidgeting, Laura is at once performing her refusal and refusing to perform. The reason I call this *unaction* and make a distinction with *inaction* is that while the latter is clearly the opposite of action, a lack or non-existence thereof, the former, I suggest, is marked not by a lack but by a refusal, a withholding, a resistance, or a deliberate absenting of action, where action is not merely missing; instead, its absence is felt and to a degree acknowledged. Action and unaction here are not opposites but corollaries, two similarly felt presences, just as a shape cut from a paper can be gleaned not just from the cut out itself but also from what is absent on the paper.

For this idea of Laura’s unaction as evoking wider political resonances of the refusal to work from feminist and disability studies, I draw from Jack Halberstam’s idea of shadow feminism. He conceives shadow feminism as:

A feminist politics that issues not from a doing but from an undoing, not from a being or becoming women but from a refusal to be or to become woman as she has been defined and imagined within Western philosophy … This feminism, a feminism grounded in *negation, refusal, passivity, absence, and silence, offers spaces and modes of unknowing, failing, and forgetting* as part of an alternative feminist project, a shadow feminism which has nestled in more positivist accounts and unraveled their logics from within. (Halberstam 2011, 124; emphasis added)

The rebellious potential of discontinuity, stillness, objectness, cesuras, and “radical passivity” (Halberstam 2011, 136) underpins Laura’s object-encounters, with the unaction of fidgeting as an interruption of the pragmatic world of typing courses and gentleman callers. Objects afford her a space to become the carer, subject, and gazer of an exhibit of glass animals and tropical flowers, the player of music on the victrola, and the creator of romantic myths with unicorns, as opposed to a fragile, cared-for, exhibited plaything, and a commodity of exchange in the marriage market, at the mercy of gentleman callers. Fidgeting as an affective gesture also creates moments of stillness, pausing dramatic action and the linear momentum of the play. Through the unaction of fidgeting, there are breaks, commas, and finally ellipses in Tom’s articulation of Laura as well as the relentless unfolding of the plot that would inevitably repeat the fates of Laura’s literary foremothers and end in either marriage like the heroines of Victorian sentimental novels or in death like those of European realism.

Let us go back to the central question I have been exploring: What exactly is Laura doing when she is fidgeting? While there are moments where she polishes and toys with the objects, unseen except by us and untainted by an emotional or psychological stirring, there is no denying that many fidgeting moments are reactionary, a frenzied response to the diatribe of “do something.” Laura winds up the victrola when her mother finds out about her typing class, “darts” to it, and “winds it frantically” (Williams 2016, 55) when asked to open the door and reaches for a piece of glass when confronted with the prospect of marriage. This reaction, where technically she does something- uses her hands, *occupies* herself, appears busy, and clearly possesses knowledge of the *occupation* of how to care for the objects- is a withdrawal from intelligible action, an action that progresses or produces. The two logical reactions to the pressure to act would be to either remain defiantly inactive or to succumb and act. Fidgeting, on the other hand, is an action that is short-circuited, rhetorical, and tautological. It does not undo, and while it is reactionary, it does not react in the sense of standing up or fighting against. This action that frustrates causality, progression, and intelligibility is then what constitutes Laura’s unaction, which is rebellious not because it drives her to actively rebel but because it changes what it means to act or rebel.

Laura’s fidgeting with her objects of care is not redemptive; it is not a lifeboat pulling her out of her circumstance, giving her purpose, or saving her from the fate of “birdlike women” (Williams 2016, 20). Instead, tangible care further anchors her to her embodied experience. It does not lift her out of her passivity and otherness but entrenches her within it, allowing her to resist canonized myths of both heterosexual and economic production and practice an alternate way of being. While I argue that Laura finds rebellious potential in her unaction, it by no means follows that she reframes productivity or changes its meaning to claim her own “action” as productive. Instead, she supplants the idea of compulsory productivity (which, as Fox points out, is deeply rooted in compulsory ablebodiedness and heteronormativity) with an absence or a shadow thereof, committing what Babcock (1999, 18) calls the “sin of inefficiency.” Laura’s continual fidgeting registers resistance to the compulsions of efficiency, normativity, legible femininity, and to the guilt of nonproduction that troubled Tom and, as Mao suggests, seeped into many modernist texts. The “rebellion” of the unaction of care is then not located in her establishing a redemptive, subversive utopia of glass in the face of oppressive forces: instead of doing the opposite or undoing, Laura’s unaction engenders a not doing, rebellious in the very fact that her rebellion (and arguably, agency and subjecthood) is not reliant on action.

## Tom’s Elusive Memory-Objects

Tom and Laura share their qualms about the production imperative. Tom finds himself unwillingly absorbed in this national myth of progress, producing shoes instead of poems, till his rebellious gesture of inscribing his art on a shoe box, a metonym of the very forces that frustrate his creative impulses, gets him fired. The play is his memory, but the slipperiness and untameability of his memory materials are evident from the very beginning, as “memory takes a lot of poetic licence” (Williams 2016, 9). On the other hand, Laura is adept at controlling the signification of her materials of care, making the semiotic possibilities of the glass “behave” in accordance with her will, and thus becoming a subject through the objectifying act of fidgeting, as observed above. Tom is desubjectivized not only by the dehumanizing forces that reduce him to a cog in a machine but also by virtue of his inability to make his materials, his memory narrative, behave. While both Tom and Laura attempt to preserve their own Romantic myth of creativity, which is pit against the national myth of productivity, Tom fails to sustain his resistance.

Laura’s act of fidgeting, when rescued from its reading as an anxious or escapist tic and recast as a practice of care, punctures Tom’s attempt to pedestalize her and cast her as a victim or a scapegoat at the altar of conventional narratives of a good life and traditional femininity. This can be seen in the arresting moment at the end of scene 3. After an argument with Amanda, Tom storms off and accidentally knocks over Laura’s glass animal, to the shock and dismay of the characters and audience alike. It might be tempting to read this moment as the object’s rebellion against Laura but ironically, this furthers their role as instruments of Laura’s resistance to the taming forces of Tom’s memory. The objects dictate the terms on which Laura herself becomes an object: of pity or paternalistic care, of Tom’s memory of her, of Amanda’s schemes, and of the marriage market. They allow Laura to regulate the pity and sentimentality the play might evoke. This scene then charts the misbehavior not of Laura’s objects of care but instead of Tom’s narrative materials, making Laura the object of our sympathy and Tom that of our derision. Pragya Gupta (2016, 162) contends that “other characters forge their own relationships with the reader/audience unencumbered by Tom’s perception of them [who] comes to be judged to the same degree as he is judging them.” While the other characters become the “materials” of Tom’s narrative, they do not recede silently into Tom’s memory without leaving a remainder.

Fidgeting allows Laura to, at times, be an object of care and pity and, at times, become a subject by caring for the objects, thus resisting victimhood and oversentimentality. As Andrzejewski argues, Laura’s silence is seen as

an invitation to narrate a more pleasing, intelligible narrative of femininity, one that is fragile, vulnerable, and dependent. Although men undeniably name and narrate throughout the play, Laura and Amanda articulate memories and cast worlds that reenvision flowers, glass, and other pretty objects. (Andrzejewski 2017, 47)

Both Amanda’s anti-narrative of memory, where she frequently recounts her romanticized past as a Southern belle, going against Tom’s representation of her as a caricature, and Laura’s use of objects that, while fragile and delicate, serve as instruments of resistance to forces of narrative taming, reveal Tom’s inability to control his narrative. Tom’s subjecthood is as tenuous as his grasp on his materials, and he continues to be a part of the “interfused mass of automatism” (Williams 2016, 9) until his final escape.

## “Don’t Say Peculiar”

The other character besides Tom, who breaks Laura’s glass animal, is Jim, the “emissary of the world of reality.” He is “the long-delayed but always expected something that we live for” (Williams 2016, 11), the Godot who finally arrives only to leave. He is the perfect spokesperson (or victim) of the American dream of self-improvement and the ardent follower of Amanda’s refrain of “Try and you will succeed!” (33). When Jim arrives in the last scene, he has extinguished Laura from memory till he exclaims, “Aw, yes, I’ve *placed* you now!” (70, emphasis added). Laura is a figure narrated and memorialized by three men: Tom, Williams, and Jim. Jim nicknames Laura “blue roses,” a mishearing of pleurisies. This act further mistakes Laura’s silence for an invitation to rewrite her identity. Laura’s earlier association with deferred flowers of the offstage glass house, the mythical unicorn, the otherworldly light of virginal angels, and now the misnaming that associates her with the transcendent “blue roses” that do not occur naturally further trap her in an identity that deems her a misfit, an anomaly, or leaves her altogether obfuscated. She is subjected to, alienated by, and *placed* within a legible version of femininity by Jim’s misnaming and Tom’s memorializing. She resists this name and, by extension, submission to men’s image of her, but this time by speaking up rather than fidgeting: “But blue is wrong for—roses” (81). Andrzejewski (2017, 45) argues, “Like the Wingfields and audiences, Jim is eager to place Laura and her beauty, when Laura is, in fact, narrating a more complicated version of her own identity.” She then risks being an object of reproach by resisting conformity to these linguistic acts of taming disguised as care and chooses to fidget with tangible objects over the world of deferred flowers, abstractions, and language.

Both Amanda and Jim embody the “prescriptions and values of organized society, and their identities cannot be separated from the conventionalized modes of behavior authorized by the Culture Industry” (Babcock 1999, 21). Jim ventriloquizes the “cruel optimism” of this system in his unwavering belief in the future of technology, while Amanda does so by peddling subscriptions to beauty magazines. Within these narratives of beauty myths and technological progress that 1930s America lets itself be soothed and comforted by, there is no place for the self-doubting or the fragile. Jim delivers an encouraging speech to Laura where he deems self-perception as the root of all her problems. This echoes Amanda’s earlier chastising response to Laura’s exclamation, “But, Mother— … I’m— crippled!” (Williams 2016, 21). Amanda forbids Laura from acknowledging her disability. When Tom suggests that she seems “a little peculiar” to outsiders, Amanda scolds, “Don’t say peculiar” (47). In suggesting that there is something unspeakable or shameful about Laura’s identity she performs, Kent (1988) points out, a powerful directive against acknowledging disability identity. Laura has been ingrained with a strong sense that she is not normal and is confronted with contradictory options in dealing with others’ reactions to her disability. While Amanda insists that Laura hide her “little defect” (Williams 2016, 21), Jim diagnoses Laura’s self-consciousness as the problem, replacing her pleurisies with blue roses. This echoes Jina B. Kim’s idea of diagnosis as a “narrative genre” with the potential to perform epistemic violence (Chen, Khúc, and Kim 2023, 9). Amanda, through her denial of Laura’s experience, Jim, through his myths of self-improvement, and Tom, through his taming narrative, all perform the diagnostic authority to “narrate disabled lives and bodies” (9).

Jim offers Laura a cure, suggesting that she need only to think of herself as superior, a blue flower among weeds. His ableist rhetoric deems perspective as the antidote to Laura’s self-consciousness. His clear disinterest in her glass indirectly places blame on her personal relationship with objects for her alienation rather than her objective, structural circumstance. He relays in his advice the cliched tropes of medical paternalism, echoing “rubrics of self-help familiar to audiences then and now: … the non-disabled person who knows better than the disabled person what they should make of their disability” (Fox 2018, 139). Amanda’s insistence that Laura hides her disability or overcome (and hence acknowledge) her condition by “develop[ing] charm” (Williams 2016, 21) and Jim’s blaming of Laura’s perspective rather than the attitudes and systems surrounding her inflict and mirror the oxymoronic forces of the societal narratives of ableism and productivity. Laura is then subjected to contradictory pressures and prescriptions to put the non-disabled person at ease: to simultaneously hide her condition and overcome it. As observed above, Laura does not experience care on her own terms, as a condescending, paternalistic brand of care is imposed on an unwilling subject, turning her into a cared-for object. As Chen explains, “One form of what is understood as dehumanization involves the *removal* of qualities especially cherished as human; at other times, dehumanization involves the more active *making* of an object” (Chen 2012, 43; emphasis in the original). Amanda, Tom, and Jim do both: *remove* her agency and ability to narrate the self (attributes associated with the thinking, self-possessed subject) and *make* her an object of care. The practice of care by the emissaries of exclusionary narratives of technology, progress, Culture Industry, beauty standards, and economic productivity becomes an instrument of dehumanization, control, and policing. As functionaries of the values of organized society, they not only consume these narratives but also police bodies and desires to promote “adherence to a normate” (Fox 2018, 136).

Jim’s dismissal of everyone as “common as—weeds” (Williams 2016, 81) is not without irony, considering his complacence with the leveling system and his cold rational approach to attaining his dream of material success, of “*Knowledge*—Zzzzzp! *Money—*Zzzzzzp!—*Power*!” (76). The inclusion of knowledge brings up questions of not only the emotional and structural harm of the opportunist world of material progress on someone like Laura and her world of unaction, but also the epistemic harm thereof. The refusal to listen to Laura articulate an identity through objects, one that does not fit into Jim’s cold optimism, the invalidation of her reality, and the forceful naming subject Laura to epistemic and linguistic pressures and leave no room for alternative ways of being. Jim’s perpetration of this stands for the larger system of standardization and homogenization outside the realist living room, where other ways of knowledge and being are placed under erasure or, worse, systematically dismantled.

## Shattering of Glass

When you look at a piece of delicately spun glass, you think of two things: how beautiful it is and how easily it can be broken.

—Tennessee Williams, *The Glass Menagerie*

The dismantling of Laura’s alternate way of being is materialized through Jim’s breaking of the glass unicorn. Jim’s reckless “operation” of Laura’s unicorn transforms it from the stuff of fables and magic to ordinary and earthly, “just like all the other horses” (Williams 2016, 79). The unicorn is replete with connotations that make it an extension, symbol, or site of Laura’s identity: like blue roses, it is otherworldly and magical and, hence, has romantic and spiritual connotations; its breaking points to the organizing, managing, and arranging of Laura’s body (physically demonstrated in the scene where Amanda dresses Laura), her physical and ideological policing to fit the mold of a commodity in the marriage market and conform to the established value systems, effectively bending her till she breaks. But less overtly and perhaps more importantly, as Bigsby (1997, 36) argues, the snapping of the unicorn’s horn “stands for something more than the end of a private romantic myth. It marks the end of a phase of history, of a particular view of human possibility.” For some critics, the snapping of the horn heralds the outside leveling world into the Wingfield home, asserting that “the national drama of progress albeit denied by the national reality of Depression is one which has no place for the fragile, the poet, the betrayed, the deserted” (Bigsby 1997, 41). Read as a fidgeting *with* Laura, this conjures an image of gripping an object until it breaks. Seen from the lens of objects, the breaking of the horn perhaps points to a breaking of the spell where the audience is complicit with Amanda, Jim, Tom, and, to some extent, Laura herself in thinking that she cannot desire more than the prescriptions of societal (and play) scripts. As Andrzejewski argues:

The Glass Menagerie depicts this kind of desire for another way of being in the world, depending on how Laura and her beauty are read … It is hard for audiences not to share Amanda’s desire for Laura to fit into conventional narratives of the good life and wish for her the only happiness offered by a poisonous, insolvent present. (Andrzejewski 2017, 44)

Laura’s fidgeting with glass remains stubbornly unintelligible and opaque, refusing to mean and be translatable or legible within conventional codes and normative modes of being. Amidst the leveling forces of standardization and mass production that homogenize men in the workforce, women in the marriage market, and objects as commodities, Laura’s objects of care defy this economy of interchangeability, if only through a refusal to mean and be comprehensible within it. Jim and Tom’s destruction of glass (albeit accidental) attempts to render transparent and intelligible—and, in so doing, demystifies—the audacious opacity of Laura’s materials of care. The destruction of the object inevitably gives rise to “melancholy and gloom,” which Bill Brown (2003, 6) connects with the “human response to the soullessness of modern life [and] the insufficiency of the desired object.” Much like the shoes that Jim and Tom consider inadequate objects of care, creation, or productive and artistic sustenance, leading them to a path of self-improvement and self-destruction, the glass, too, proves unsatisfactory in Jim and Tom’s search for *more.* The two men’s destruction of the glass unicorn reveals an absence of meaning or the idea it holds for Laura as it is “everywhere and nowhere” (Brown 2003). Laura’s unaction of fidgeting and material care conducts her resolve to remain unexplainable; the gesture stubbornly refuses to make sense or be accessible and reflects not her “inability” but the lack of desire to “standardize herself” (Babcock 1999, 25). While the position of shoes and the glass unicorn in the play is quite different ideologically, they share their role as objects that reveal the two men’s misguided quest for a source of meaning. Meaning, for them, is something that emanates from a source that they can aspire to reach, grasp, or acquire rather than something that is mutually created, nurtured, and toyed with but forever deferred. Their tendency to demystify and thus possess the elusive dictates their own lives in their ambition to conquer and tame the great unknowns of technology and the sea.

While glass has been shattered before, during Jim and Laura’s dance scene, its fragility is enhanced as it is planted within the corporeal and bodily field of touch, movement, and intimacy. The exchange prologuing this moment where the two characters discuss the glass collection at length ensures that we remember its presence. A prolonged discussion about the fragility of an object and its importance to character, along with moments of care with the touching and cherishing of an object, carries with it the premonitions of destruction; it might make us predict the shatter, given our familiarity with traditional narrative (novelistic, dramatic, cinematic) tropes and trajectories.

There is a direct visual and affective juxtaposition or even confrontation drawn between the objects and the bodies that threaten them. But the two are also similarly otherworldly: the dance has an air of an orchestrated dream that Laura gets to momentarily indulge in, a fantastical world she steps into, like the one inhabited by the unicorn. There is a slight hope fostered by this pseudo-fantastical atmosphere that maybe the glass will not break; maybe this fantasy will sustain itself; maybe the association between fragility, care, and destruction will be broken. There is also a sense that the affinity drawn between the two otherworldly presences and intimacies (between the dance and the glass unicorn) might be mutually sustainable. But these prove to be mutually destructive as both the figments of fantasy are made cruelly real.

Both the fairy-tale moment and the glass are shattered in a single sweep of Jim’s indiscriminate movement. Just as Laura’s silence and apparent passivity are read as an invitation to breach it, so do the nurturing and caressing of objects invite an omen of destruction as Williams himself suggests in his production notes mentioned in the opening of this section. The very presence of stillness, fragility, and passivity associated with an object of care then evokes an ontologically indiscriminate attempt to transgress and break it (be it Laura or the glass). The moment of shattering then lies somewhere between phenomenally jarring and predictable.

## The Afterlife of the Glass Unicorn

Much like the Chekhovian gun, fidgeting on stage carries prolepsis of destruction and, by extension, a destructive end. The breaking, I suggest, does not imply the triumph of the leveling forces of the real world but instead a reflection and scrutiny of these forces. The prescriptions that circumscribe Laura to the possibilities penned by the Jims of the world emerge as inadequate, as the breaking of the horn cues the reader/audience to look at Jim and the worldview that he represents with less understanding eyes and even outright blame (as earlier with Tom). This degree of distancing and reflection on the system that produces Jim and allows him to flourish is accompanied by the breaking of the readers’ trust in the narrative of a good life offered to Laura and a reflection on our own yearning for the fairy-tale ending promised by this narrative. This potential of the snapping of the horn is only available to us when fidgeting is rescued from an exclusively semiotic or psychoanalytical frame of a symbol or a nervous, anxious act and observed through the ideas of care, unaction, and destruction as a prominent affective gesture.

The question of whether the materially packed ending marks Laura’s growth or succumbing to what can be called the camp of productivity, confidence, and self-improvement (celebrated by Jim and Amanda) has been a point of debate among critics. One way of reading the end is through the network of objects and gestures that the last scene orchestrates: while she raises Jim’s hand and gives him the broken unicorn (now just a horse) and smiles, she also fiddles with the victrola and blows out the candle (on Tom’s cue), leaving the stage and the audience enveloped in darkness. Parting with the broken unicorn can be seen as Laura parting with remnants of her difference from the industrial-patriarchal nexus (being a unicorn in a world of horses) and the creative and romantic impulses that she managed to preserve from the outside forces through her practice of material care. On the other hand, in light of the deliberate inclusion of fidgeting after the parting, this gesture must instead be read as Laura parting with a symbol of her victimhood and of the leveling system, marking its failure to infiltrate her world of unaction. As the glass crosses the border of the two worlds, taken away by someone willing to play by the rules of the exploitative system, the act of fidgeting and its associated mythology of care infiltrates Jim’s world and his rhetoric of progress and material success. Laura continues fidgeting with the victrola and, we can safely assume, the rest of her menagerie. Furthermore, while it may be tempting to read the blowing of the candle as cued by Tom, we must remember that this is his memory. Laura’s act must precede Tom’s narration as he merely captions it for our benefit. Laura is then released from the masculine forces of Tom’s memory through her fidgeting and snatching of the reins of the narrative, inherited by her as Tom leaves. Fidgeting and material care frustrate Tom’s attempt to make the play a *künstlerroman* or a male adventure narrative that finds no place within the play, which is now Laura’s “material” and must exist in a space outside the door of the realist living room.

Despite this reading, we are nonetheless left with no suggestion of what the larger schema of the characters’ lives is and from where the play has been plucked. Is it a slice of their larger lives that will continue replaying a version of the action in perpetuum until Tom finally leaves? Does the end mark a radical change and break from a pre-existing cycle or was the action unique and uncharacteristic, briefly making the Wingfields worthy subjects of a play? What happened to Laura’s walks, to her now chipped menagerie, and to Amanda’s search for a gentleman caller? Does the closing silent dialogue, seemingly a moment of consolation and love between mother and daughter that we do not hear, mark a shift in the Wingfields’ relationship? The epilogue of fidgeting toys with loose ends and incompleteness, harnessing its earlier associations with interruptions, silences, and residues that push against the forces of realist, closure-orientated narratives and male storytelling. These loose ends challenge not just the comfort of a realist closure but also Tom’s fashioning of himself (seconded by critics and reviews) as a Shelleyan poet-prophet, an “unacknowledged legislator … of the world” (Shelley 2002).

## Conclusion

In this light, there is a suggestion that we can yearn for a different future for Laura that may not be possible in the world of the play but is certainly thinkable. Like Laura’s glass unicorn, the illusion of a happy ending heralded by Jim must break. The glass then needs to break to shatter the taming forces of narrative and the illusion of Laura finding a home in the conventional marital plot. The shattering of this illusion allows her to yearn for a space beyond, perhaps in the offstage space beyond the realist door*,* even if that space is not available historically in America of the 1940s.

In a world where men are marked by absence, where fathers and sons abandon the home for quixotic adventures and gentlemen callers come and go, the presence-asserting objects that anchor women in an alternative *now* become an antidote to the taming flow of time, narrative, and progress. This reconsideration of the play does not attempt to “rescue” Laura from her passivity but to suggest the rebellious potential of her unaction. This potential is only available when we move beyond the anthropocentric, subject-oriented approach to the play. Laura’s fidgeting with objects highlights the inadequacy of narratives of male storytelling, normalcy, productivity, and compulsory heterosexuality to accommodate her alternate way of being. While conventional readings dismiss the objects in the play as anchors pulling the characters, especially Laura, down, I posit these objects as sponges, absorbing and exuding personal brands of care and/as agency. These alternate hermeneutic possibilities emerge when fidgeting is rescued from critical dismissal as an escapist gesture or a psychoanalytical subject and read instead as a practice of care. Beyond a literary gesture of material care, I have attempted to approach fidgeting as a refusal, an opting out, or a “prefer[ring] not to” (Melville 1948, 24). This draws from the radical potential of passivity and refusal in feminist and disability discourses as a rebellion against ableist heteronormativity and action-based determination of subjecthood and value. Seen as an affective and ideological orientation towards objects, drawing sensory practices of care, stillness, fragility, objectness along with the underpinnings of work, productivity, and gendered care, fidgeting becomes a fertile ground to interrogate the modernist material imagination planted within the specific valences of artistic and economic productivity in mid-century America.

Objects of care help Laura perform alternate forms of agency and recognize (if not redress) the exclusionary configurations of established value systems and dominant ideology. Laura’s practice of care, unaction, and objectness allows her to withdraw from the othering definitions of normalcy. They redefine her as a different kind of carer, questioning others’ practice of paternalistic care and problematizing her fictional and critical romanticization or dismissal as a plaything. Fidgeting further anchors Laura in her difference and engenders her alternate way of being; it refuses to be a redemptive act that rescues her and instead makes her stubbornly unintelligible and impenetrable. When read through her relationship with objects, she emerges as an anachronistic character, at once romantic and absurd, trapped in a modern realist narrative and thus “peculiar” for her paradoxical way of being. Laura’s fidgeting and material care then register not her inability to find a place in the conventional scripts penned for her by Williams, her family, society, and even the audience but the failure of these scripts to care for her.

## Declarations

### Endnotes

^1 ^All references to *The Glass Menagerie* will be from the 2016 Worldview edition (edited by Payal Nagpal).

^2^ New American Stagecraft was a scenographic movement that used ideas from European modernism for American theater practiced by Robert Edmond Jones, Lee Simonson, Jo Mielziner, among others.

^3^ While scholarly attention has been given to touch, sensory experience or habit in literature and drama (see Abbie Garrington, *Haptic Modernism*, James Krasner’s *Home Bodies*, Joe Moshenska’s *Feeling Pleasures*, among others), they tend to overlook the specific subject of fidgeting.

^4^ Lauren Berlant (2011, 1) argues that “A relation of cruel optimism exists when something you desire is actually an obstacle to your flourishing.” She calls this optimism “stupid” in its “faith that adjustment to certain forms or practices of living and thinking—for example, the prospect of class mobility, the romantic narrative, normalcy, nationality, or a better sexual identity—will secure one’s happiness” (126).

^5^ Here, I am paraphrasing the famous refrain (“I would prefer not to”) by Melville’s character Bartleby, whose resistance to the work imperative can be read as a precursor to Laura’s (Melville 1948, 24).

^6^ Bigsby (1997, 32) explains Williams’s qualms with “the Cinderella story, with its account of moving from rags to riches, as a primary and destructive American myth, for it is the fate of his characters, and particularly those in his first Broadway success, to miss life’s party, to be left with no more than the ashes of a once-burning fire.”

^7^ Discussing the intersection between disabled and queer positionalities, gender discourses, and anti-work politics, Chen, Khúc, and Kim (2023, 3) ask, “What political traction does the refusal of work hold for those who have typically been refused as workers?”

^8^ Mao argues that despite high modernism’s (specifically Bloomsbury’s) resistance to the “commodification of the aesthetic,” their practice is nonetheless marked by certain guilt and justification of nonparticipation in the “production imperative.” He further points out that despite a deep mistrust of the imperative of production, modernists found it “as profoundly suspect as seductive” (Mao 1998, 21).

^9^ This is reminiscent of Adrienne Rich’s (1979) argument that the act of naming has traditionally been “a male prerogative.”

^10^ I am using Jacques Derrida’s poststructuralist ideas of the endless deferral of meaning of a sign and the modernist deconstruction of the transcendental signified to highlight the men’s aspirations that parallel this deconstruction (see Derrida 1982).

^11^ While critics like Bigsby, Kent, and Levy hold that the play is a vivid story of Laura’s wasted life, Andrzejewski, Cardullo, and Gupta identify resistant possibilities of the end.
